# Simplifying informed consent as a universal precaution

**DOI:** 10.1038/s41598-024-64139-9

**Published:** 2024-06-08

**Authors:** Iris Z. Feinberg, Ajeet Gajra, Lori Hetherington, Kathryn S. McCarthy

**Affiliations:** 1 Department of Learning Sciences, College of Education and Human Development, Georgia State University, Atlanta, GA USA; 2 Hematology Oncology Associates of CNY, East Syracuse, NY USA; 3 School of Public Health, Georgia State University, Atlanta, GA USA

**Keywords:** Health services, Health care

## Abstract

One barrier to participating in clinical research is that patients with low literacy skills (1 in 5 US adults) may struggle to understand the informed consent document (ICD). Writing consents using health literacy and plain language guidelines including simplified syntax and semantics can increase understandability and facilitate inclusivity of research populations with literacy challenges. Our study aim was to evaluate a simplified ICD for understandability while considering factors known to relate to comprehension (reading skills and working memory). We performed an on-line survey of 192 adults ages 18–77 in Georgia. Participants performed significantly better on the simplified ICD test. We built an additional model with all version x measure interactions (i.e., age, sex, race, urbanicity, GMVT, WM). This model did not significantly improve model fit, *F* < 1.00, suggesting that individual differences did not moderate the effect of simplification. Our findings suggest that using plain language and simplified syntax and semantics in ICD as a universal precaution may reduce cognitive reading burden for adults regardless of differences in reading skill or working memory. Increasing understandability in ICD may help improve targets for clinical trial enrollment.

## Introduction

Clinical trials provide the backbone to evidence-based medicine, providing answers to the safety and efficacy of life-saving medical and behavioral interventions. Patient enrollment is the key to successful clinical trials, however, data show that more than 80% of US clinical trials fail to meet enrollment timelines^1^. There are many reasons why people do not participate in clinical trials including burdensome study requirements, lack of understanding about the process, fear of randomization, and distrust of researchers^2–5^. The National Institutes of Health obligated $28 billion for clinical trials between 2017 and 2021^6^, but more than half of all clinical trials worldwide are terminated due to a low patient accrual rate^7,8^. In addition to wasted funds and system-wide inefficiencies where clinical trials are implemented (e.g., staffing, training, recruitment costs), there are also ethical issues for enrolled patients whose participation may not contribute to scientific knowledge^9–11^. Further, within enrolled patients, racial and ethnic minorities are frequently underrepresented. As a result, scientific findings and medical advances can be skewed and ungeneralizable, causing greater health disparities and less benefit among diverse populations^1,12,13^.

A critical barrier to participating in research is that participants need to be able to read and understand informed consent documents (ICD)^14–17^. ICD describe the study and its treatments or intervention, risks and benefits, patient obligations, and confidentiality, and are both an ethical and legal requirement for participation in clinical trials. Most ICD require a high level of reading skill and comprehension and are often written at a level that is too difficult for many patients to comprehend^14,15,18^. As medical treatments continue to progress, ICD procedures have grown in complexity as well. Cancer is a complex disease that has a major impact on society; approximately 40% of people will be diagnosed with cancer during their lifetime and as of 2020, an estimated 117 million people were living with cancer in the US^19^. Types of clinical trials include studying treatments, prevention, screening, and supportive care, and are always initiated by patient review of an ICD which can be extremely complicated, lengthy, and difficult to read. The goal of the ICD is to guarantee patient comprehension and autonomy through sufficient explanations of different therapeutic options, effects, risks, and potential side effects. However, a result of more complex clinical trials is that ICD often becomes overly long, dense, and complex^14^.

Complex and difficult to understand ICD may pose a threat to the recruitment goals of clinical trials. More than 1/3 of U.S. adults struggle to understand basic health information^20^. Referred to as a person’s health literacy, these lower skills hamper a person’s ability to find, understand, and use health information to make informed decisions. Only 12% of US adults are considered proficient with a high enough level of health literacy to engage in complex medical discussions and decision-making including understanding clinical trials and informed consent^21,22^. Lower health literacy is more prevalent in disadvantaged population groups including racial and ethnic minorities, older adults, and those with low educational attainment^20^. Although anyone can have low health literacy at any time depending on the situation (e.g., are afraid, anxious, fatigued, sick, or have just gotten a new diagnosis)^23^ the high prevalence of disadvantaged populations who have low health literacy may mean that people who are representative of those populations may not sign up for clinical trials^20,22^. Thus, improving understandability of ICD may improve participant accrual and facilitate inclusivity of diverse research populations with health literacy challenges^4,5,7,12,16,17,24,25^.

The informed consent process is a founding principle of research ethics and goes beyond simply reading and signing a document^11,16,26^. The prime purpose of informed consent is to make sure a participant understands the process, associated benefits, and risks, and their understanding has led to permitting participation in the research. Only then is the consent given valid. Inadequate comprehension may lead individuals to sign ICD without understanding basic research concepts such as risk, randomization, or treatment options^27^. Participants asked to decide without demonstrating understandability leads to violating core ethics of human subjects’ research and respect for personal autonomy^26^.

Although the National Institutes of Health recommends that all health materials be written at the 7th to 8th grade level^24^, most ICD are written above the 10^th^ grade level above^16,18,27,28^ which makes them too challenging for the average reader. About half of US adults read at the 8th grade level; Black and Hispanic U.S. adults are substantially over-represented in populations with low reading skills^21,29^. Improving health materials includes simplifying text to improve readability so that readers can better comprehend what is written and grasp the message the first time they read it^12,24,30^. One method of text simplification is using plain language principles. The U.S. government describes plain language as communication that is understood the first time it is seen or heard, describing it as “easy to read, understand, and use”^31^. Plain language guidelines include simpler word choice (semantics), simplified sentence structure (syntax), the use of shorter sentences, using active voice and present tense, and focusing on what readers want to know^31^. Theories of text comprehension suggest that a readers’ understanding of information in a text is driven by features of the text itself as well as aspects of the reader^32^. These theories emphasize that reading comprehension draws upon domain-general abilities, such as working memory^33^ as well as domain-specific reading skills^34,35^.

This study was a pilot study aimed at exploring the utility of plain language revisions that attended to multiple dimensions of linguistic complexity. More specifically, the aim of this study was to assess the difference in comprehension between an original cancer clinical trial ICD and the same ICD simplified using plain language guidelines including simplified semantics and syntax. Participants were presented with a written ICD and asked to assess if statements were either true or false based on their reading of the ICD without the benefit of discussion with a researcher. Participants also completed reading skill and working memory measures to explore how differently skilled readers might respond differently to these changes. Our research questions were:

1. Does a simplified ICD improve comprehension relative to the original version?
2. Does comprehension, and differences in comprehension between the original and simplified ICD, vary across demographics (age, sex, race, urbanicity) or individual differences related to comprehension (reading skill, working memory)?

## Results

We simplified four sections of a colorectal cancer clinical trial informed consent by using plain language guidelines (see “Methods”). Both original and simplified versions were submitted to Flesch Kincaid readability analysis (FKGL) and VT Writer analysis^36^. The original ICD had a FKGL of 12.3. By contrast, the simplified version had a FKGL of 8.2. VT Writer long sentences and passive voice for the original ICD were 15.5% and 8.6%, respectively; for the simplified ICD scores were 7.4% and 5.3%, respectively. See Table 1 for a sample section of the original and simplified ICD.

**Table 1:** Original and simplified ICD section (What is the purpose of this study).

Text version	Number of Words	Flesch kincaid Grade Reading level	Long sentences (% of all sentences)	Passive voice (% of all sentences)
Original	416	12.3	31.3%	27.3%
Simplified	203	7.5	0.0%	0.0%
Original text				
We invite you to be in a research study (also known as a clinical trial). The trial is a blend of a novel therapy called CB-839 and standard-of-care treatment of panitumumab (VECTIBIX). We are asking you to take part in this research study because you have colorectal cancer (CRC). The purpose is to test how well the drugs CB-839 and panitumumab work together to keep your cancer from growing and spreading. Glutamine is a molecule that plays many roles that cells require for survival. Compared to normal cells, some cancer cells have increased glutamine metabolism that drives their growth and progression. The study drug CB -839 works by blocking a key regulator of glutamine metabolism. Some cancer cells have too much epidermal growth factor receptor (EGFR). EGFR is a protein on the surface of cells that helps your cells grow and divide. Having too much EGFR can cause cancer cells to grow faster. The study drug panitumumab works by blocking the EGFR to stop the growth and spread of cancer cells. Panitumumab is currently FDA-approved to treat certain types of CRC. This document describes the following to help you make an informed decision to be in the study: informed decision to participate: the purpose of the study, The procedures needed for the study, the possible benefits, and risks of being in the study. Please take time to read the following information. Feel free to talk with your doctor, nurse, family, or friends before deciding. If you have questions, you may ask the study doctor, Dr. XXX, for more information. For this study, we ask you to undergo a pre-treatment tumor biopsy and two positron emission tomography (PET)-CT scans. The biopsy and PET-CT scans will measure the amount of glutamine metabolism in your cancer. We also ask you to provide blood to look at circulating markers to explore how they affect health and disease. XXX is sponsoring this study. The company Calithera is providing the CB-839. We will obtain Panitumumab as standard of care from commercial supply. The doctor in charge of this study is XXX. About 29 participants from XXX will take part in this study				
Simplified text				
We invite you to be in in a research study (also called a clinical trial) that combines a new drug with the drug you usually get as part of your treatment. The new drug is called CB-839 and your usual drug is called panitumumab (also called Vectibix). You can be in this research study because you have colorectal cancer. These drugs block different parts of a cancer cell’s growth. The 2 drugs have not been used together to treat your disease. The purpose of this study is to test how well the combined drugs work to keep your cancer from growing and spreading. This document describes the purpose of the study, your rights and responsibilities, the procedures needed by the study, and the possible benefits and risks of being in the study. Feel free to talk with your doctor, nurse, family, or friends before deciding. The doctor in charge of this study is XXX. The study is being sponsored by XXX. The company that makes CB-839 will supply the drug. We will get Panitumumab (also called Vectibix) from the usual commercial supplier We will ask 29 people with colorectal cancer to be in the study				

Mean age of participants was 36.9 (*SD* = 14.46; range = 19–77), 60.4% identified as Female. Almost 50% of our sample was White (49.5%) and 85.8% were from urban zip codes in Georgia. To account for relevant individual differences, participants also completed the Gates MacGinitie Vocabulary Test (GMVT)^37^ as a proxy for reading skill, a proxy measure for working memory (Woodcock Johnson Numbers Reversed test; WJNR)^38^, and a demographic survey that included age, sex, race, and urbanicity. See Table 2 for details.

**Table 2:** Demographics and cognitive variables descriptive statistics and correlations.

Variable	*N*	%	Range	*M*	*SD*	1	2	3
Age	192		19–77	36.9	14.46			
Sex								
Female	116	60.4						
Male	76	39.6						
Race								
White	95	49.5						
Black	84	43.8						
Other	13	6.8						
Urbanicity								
Urban	163	85.8						
Rural	27	14.2						
1. GMVT			0–45	22.10	11.87			
2. WJ numbers reversed			0–62	18.68	9.55	0.27**		
3. Comprehension (original)			0–15	9.15	2.89	0.42**	0.32**	
4. Comprehension (simplified)			0–15	10.53	2.51	0.43**	0.30**	0.72**

**p < 0.05.

Descriptive results by consent section (Purpose, Cost, Side Effects, Stop) are shown in Table 3. Preliminary analyses showed no differences in effects across the sections. Thus, we elected to use overall comprehension test score as our dependent variable to simplify interpretation of the results. To address RQ1 (the effect of simplification on readers’ comprehension of ICDs), we conducted a paired-samples t-test which confirmed that the simplified version of the ICD led to improved comprehension test performance, relative to the original IDC, *t*(191) = 9.36, *p* < 0.001, Cohen’s *d* = 0.68. Over half of participants (52.6%) showed improvement in comprehension when reading the simplified informed consent; 32.6% showed no improvement and 15.1% showed a decrease in correct answers.

**Table 3:** Descriptive scores by sections of informed consent.

Sub-score	Possible	Original		Simplified	
		*M*	*SD*	*M*	*SD*
Purpose of study	6	3.51	1.31	3.66	1.46
Costs to patient	2	1.21	0.71	1.32	0.74
Side effects	4	2.71	0.93	2.81	0.97
Stop being in study	3	1.72	1.09	1.90	1.07

To explore these effects more deeply and to consider the effects of demographics and individual differences, we submitted the data to a series of regression models. Model fit statistics and coefficients are shown in Table 4. In the baseline model (m0), we included reading skill and working memory z-scores as well as demographic information (age, sex, race, urbanicity). This baseline model explained 26.7% of variance in comprehension test performance, with age, sex, reading skill, and working memory as significant predictors of comprehension test performance, but no effects of race or urbancity (Table 4). The positive coefficients for age, GMVT, and working memory indicate that age, reading skill, and working memory all positively predicted comprehension test performance. Follow-up analyses revealed that women (*M* = 10.21, *SD* = 2.83) outperformed men (*M* = 9.28, *SD* = 2.64).

**Table 4:** Linear regression models.

	Baseline model (M0)				M1				M2			
	*F* = 22.68, *p* < 0.001				*F* = 34.62, *p* < 0.001				*F* = 0.23, *p* = 0.96			
	Estimate	Std. error	t-value	p-value	Estimate	Std. error	t-value	p-value	Estimate	Std. error	t-value	p-value
Intercept	8.02	0.51	15.72	0.00	7.32	0.50	14.56	0.00	7.42	0.70	10.67	0.00
Age	0.05	0.01	5.02	0.00*	0.05	0.01	5.24	0.00*	0.05	0.01	4.05	0.00*
Sex	−0.59	0.26	−2.28	0.02*	−0.59	0.25	−2.38	0.02*	−0.67	0.35	−1.89	0.06
Race	0.10	0.21	0.45	0.66	0.10	0.20	0.47	0.64	−0.06	0.29	−0.22	0.82
Urbanicity	0.27	0.36	0.74	0.46	0.27	0.34	0.78	0.44	0.36	0.49	0.73	0.47
GMVT (z-scored)	0.73	0.14	5.28	0.00*	0.73	0.13	5.51	0.00*	0.78	0.19	4.12	0.00*
WM (z-scored)	0.46	0.13	3.57	0.00*	0.46	0.12	3.72	0.00*	0.52	0.18	2.93	0.00*
Consent form version					1.39	0.24	5.88	0.00*	1.20	0.98	1.22	0.22
GMVT × version									−0.10	0.27	−0.36	0.72
WM × version									−0.11	0.25	−0.44	0.66
Gender × version									0.16	0.50	0.32	0.75
Age × version									−0.01	0.02	−0.51	0.61
Race × version									0.32	0.41	0.78	0.44

*p < 0.05.

In model 1, we added the informed consent form (original, simplified). The inclusion of this variable significantly improved model fit, increasing variance explained to 32.9%. Consistent with t-test analysis, participants performed better on the comprehension test after the simplified ICD as compared to the test after the original ICD (Table 4). This result reveals that the effects of simplification were significant, above and beyond the contribution of individual differences.

To address RQ2 (to what extent do the demographics and individual differences influence the effects of simplification?), we built an additional model with all version $\times$ measure interactions (i.e., age, sex, race, urbanicity, GMVT, WM). This model explained 33.2% of the variance, which did not significantly improve model fit, *F* < 1.00, suggesting that individual differences did not moderate the effect of simplification.

## Discussion

Our findings suggest that reducing ICD complexity by using plain language and simplified syntax and semantics may improve comprehension for adult readers. Consistent with research in reading comprehension, both reading skill (as measured by general vocabulary) and working memory predicted comprehension performance. However, there were no moderating effects of these variables. That is, simplifying the ICD was equally effective for readers, regardless of their reading skill or working memory and across age, sex and urbanicity.

While not a panacea for low clinical trial enrollment, eliminating reading and comprehension barriers to ICD may be an important first step to improving participation, and should be considered as a universal precaution^12,23,24,29^. Further, for people of color who are over-represented in cohorts with lower literacy skills^20^ and under-represented in enrollment for clinical trials^39^, ICD that are simpler to read and understand may help improve understanding, which may lead to improved clinical trial enrollment of racial and ethnic minorities. It is of note in our study that comprehension varied across sex and age, but that the effects of simplification did not vary across other demographic groups of race or urbanicity.

Our cohort may not reflect the demographics of people diagnosed with colorectal cancer or participating in colorectal clinical trials. Average age of our cohort was 37; 89% of new colorectal cancer is diagnosed in an age group of over 50 years old^40^. Although the incidence rate of diagnosis increases with age, colorectal cancer (CRC) is one of the most common cancers diagnosed for adults younger than 50 years old^40^ with an estimated increase of more than 140% by 2030. Age is an important factor in clinical research as treatments may interact differently with underlying health conditions or with medications commonly taken by people in different age groups. The incidence rate for colorectal cancer diagnosis shows proportionately more Blacks (45.7/100,000) than Whites (38.6/100,000); our study cohort had more Whites (49.5%) than Blacks (43.8%)^19^. It was not our intent to match cohorts in this pilot study. For a variety of reasons, people enrolled in trials tend to be younger and less racially/ethnically and geographically diverse than people diagnosed and seen in clinics^41^.However, these findings present an important first step in the research process and future work should examine more authentic implementation and populations.

Although some studies suggest specific skills and knowledge (e.g., topic knowledge) are more closely related to comprehension outcomes than readability^42,43^, however, at a minimum, readability promotes a better understanding of the information provided in ICD. Using plain and simplified language can lower cognitive demands, which may facilitate the inclusivity of research populations with general literacy and health literacy challenges^5,7,10,24^. Failing to include a diverse study population, can reduce scientific accuracy and generalizability of clinical trial findings, and can lead to unequal access to research benefits, harm from use of ineffective treatments, and continued mistrust of clinical research^13,17,24,25,45^. Supporting participant needs with comprehensible information developed using health literacy guidelines can provide an ethical and practical solution to address disparities in clinical trial recruitment^11,13,24,25^.

While the focus of this study was on text simplification, there are certainly other factors that can impede comprehension. For example, electronic consent (e-consent) is gaining popularity as an efficient approach to obtaining participant consent and reducing logistical challenges of paper ICD (e.g., printing, storage, etc.)^44^. Some digital literacy skills are required to ensure potential trial participants are familiar and facile with using the technology; digital health literacy includes not only the ability to find, understand, and appraise health information from on-line sources, but the necessary skills to use digital tools in the health environment^45^. Extra attention should be paid to incorporating not only easier to read and understand words, but to important web design concepts like how to display content clearly on an electronic page, using bullets and short lists, meaningful headings, larger font, and white space^45,46^.

For many people who are racial or ethnic minorities, contextual and cultural factors such as mistrust arising from a history of systemic racism in the medical system for many African Americans or linguistic challenges experienced by non-native English-speaking adults^3,8^ should be considered as important health literacy guidelines when revising ICD. The national standards for Culturally and Linguistically Appropriate Services (CLAS) in health and healthcare were designed to advance health equity and improve quality by providing a blueprint to improve patient communication, satisfaction and safety through equitable, understandable, and respectful care and services^46^. In particular, the Communication and Language Assistance Standard ensures that people can understand the health care and services they receive and can make informed decisions based on their cultural and linguistic knowledge and abilities^46^. Among other things, culture impacts how people perceive health and wellbeing and who should participate in health-related decisions^47^. Further, the language of health and healthcare is complex and jargon-laden, especially when considering research terminology (e.g., randomization, placebo) that may be unfamiliar^12,30^. Providing health information in a person’s first language can increase knowledge, however, simply translating ICD is not adequate; even plain language phrases and words may not mean the same in-language as in English. Effective health literacy strategies to improve comprehension must include formative research with the intended research population to ensure cultural and linguistic ICD compatibility^12,24,47^.

This pilot study demonstrated the utility of improving the comprehensibility of ICD. However, that are several limitations that need to be considered when reviewing the data and findings. Our aim was to understand the effect of a plain language ICD relative to the more common, complex wording of an original ICD. The within subjects’ design allowed for direct comparison of the two versions, but all participants first saw the original consent form and then the simplified version. This design leaves open the possibility of order and practice effects, such that seeing the test twice led to improved performance. Future work should better isolate the effects of plain language revision via counterbalanced presentation. It would also be of value to explore the extent to which these effects generalize to other oncology materials and/or to other types of clinical trials. One such approach would be a block randomized cross-over controlled design in which participants would view blocks of the same type (e.g., Original, Revised) of consent forms, with these blocks randomized and counterbalanced across participants. Of course, such an approach requires additional time and resources and could introduce other methodological complexity (e.g., fatigue effects, potential for spillover from one text to the next). Regardless, additional replications and increased rigor are important next steps. Consistent with the limited time we had with participants, we elected to collect standardized general individual differences (vocabulary, working memory) known to relate to comprehension. Of course, other individual differences, such as prior knowledge, are likely to affect both comprehension and the effects of text complexity and text simplification^48,49^. Future work should consider how a readers’ baseline understanding of cancer, clinical trials, or healthcare in general may impact their understanding and the effects of simplification.

We assume that improved comprehension may have positive effects on trial enrollment given that patients will have better understanding of the study and its risks and benefits. However, we did not collect a behavioral measure to directly evaluate if ICD simplification would change the participants decision to participate. We also did not assess cultural and linguistic preferences of study participants when reading ICD. Future work should more explicitly test these constructs.

## Methods

In July 2023, we randomly selected one cancer clinical trial ICD from the clinicaltrials.gov website using the following criteria: cancer, adults, drugs, studies conducted in English from 2000 to 2022. We selected four sections (Purpose of Study, Costs to be in Study, Side Effects, Stop Taking Part in Study) and analyzed them using Flesch Kincaid Grade Reading level and Visible Thread Writer (VT Writer) for long sentences and passive voice. Flesch-Kincaid Grade Level (FKGL) takes into approximates lexical and syntactic complexity via a calculation of number of syllables per word and words per sentence multiplied by a constant to produce a grade level range and are appropriate to measure written passages like sections of the ICD (DG). VT Writer is an automated language analysis platform that scores both word documents and pdfs for elements that contribute to ease of reading^35^. A health literacy/plain language expert manually simplified the ICD sections by using plain language guidelines. These guidelines include write for your audience, state major points first, be concise, write in active voice, use short sentences, simplified semantics (word choice), and simplified syntax (sentence structure). Two medical oncologists familiar with clinical trials reviewed the simplified sections to ensure the revisions accurately and completely reflected what was in the original informed consent; each oncologist concurred that the simplified version was accurate and complete.

To evaluate readers’ comprehension of the content, we created 15 true/false comprehension questions based on the information in the four sections of the clinical trial ICD. See Table 2 for comprehension questions and scoring.

In November 2023, we recruited 192 adults who live in Georgia through Qualtrics Research Services. The study was completed asynchronously. Participants first completed the demographics. They then read the original informed consent and completed the first set of comprehension questions. Participants then completed the GMVT and WJNR. Finally, they read the simplified informed consent and completed the comprehension questions. This study required ethical approval which was obtained from the Georgia State University Institutional Review Board. All methods were performed in accordance with the relevant guidelines and regulations.

Analyses were conducted in R. Descriptive statistics included means, standard deviations, frequencies, and chi-square calculations. Pearson correlations, t-tests and linear regression models were also utilized.

### Supplementary Information

Supplementary Information.

## Data Availability

The datasets generated and analyzed during the current study are available from the corresponding author on reasonable request.

## References

[1] Strasser JE, Cola PA, Rosenblum D (2013). Evaluating various areas of process improvement in an effort to improve clinical research: Discussions from the 2012 Clinical Translational Science Award (CTSA) clinical research management workshop. Clin. Transl. Sci..

[2] Mao JJ, Tan T, Li SQ, Meghani SH, Glanz K, Bruner D (2014). Attitudes and barriers towards participation in an acupuncture trial among breast cancer patients: A survey study. BMC Complem. Altern. Med..

[3] Des Jarlais G (2005). Factors affecting participation in a breast cancer risk reduction telephone survey among women from four racial/ethnic groups. Prevent. Med..

[4] Lawsin CR, Borrayo EA, Edwards R, Belloso C (2007). Community readiness to promote latinas’ participation in breast cancer prevention clinical trials. Health Soc. Care .Commun..

[5] Nipp RD, Hong K, Paskett ED (2019). Overcoming barriers to clinical trial enrollment. Am. Soc. Clin. Oncol. Educ. Book.

[6] U.S. Government Accountability Office. *National Institutes of Health: Better Data will Improve Understanding of Federal Contributions to Drug Development*. https://www.gao.gov/products/gao-23-105656 (2023).

[7] Desai M (2020). Recruitment and retention of participants in clinical studies: Critical issues and challenges. Perspect. Clin. Res..

[8] Williams, R. J., Tse, T., Di Piazza, K., & Zarin, D. A. Terminated trials in the clinicaltrials.gov results database: Evaluation of availability of primary outcome data and reasons for termination. *PLOS ONE*. https://doi.org/10.1371/journal.pone.0127242 (2015). 10.1371/journal.pone.0127242 PMC4444136 26011295

[9] Zhang E, DuBois SG (2022). Early termination of oncology clinical trials in the United States. Cancer Med..

[10] Fogel DB (2018). Factors associated with clinical trials that fail and opportunities for improving the likelihood of success: A review. Contemp. Clin. Trials Commun..

[11] Malmqvist E, Juth N, Lynöe N, Helgesson G (2011). Early stopping of clinical trials: Charting the ethical terrain. Kennedy Instit. Ethics J..

[12] Caballero A (2021). Addressing health literacy as a foundation for effective and equitable health communication. J. Consumer Health Internet.

[13] Washington V, Franklin JB, Huang ES, Mega JL, Abernethy AP (2022). Diversity, equity, and inclusion in clinical research: A path toward precision health for everyone. Clin. Pharmacol. Ther..

[14] Tilch M-K, Schranz M, Moringlane A, Theobald M, Hess G (2022). Struggling with extensive informed consent procedures for cancer trials—Is there even a benefit for the patients?. Supp. Care Cancer.

[15] Malik L, Kuo J, Yip D, Mejia A (2014). How well informed is the informed consent for cancer clinical trials?. Clin. Trials.

[16] Munley B, Buser AT, Gaudreau S, Breault JL, Bazzano LA (2018). An analysis of informed consent form readability of oncology research protocols. J. Empir. Res. Hum. Res. Ethics.

[17] Nathe JM, Krakow EF (2019). The challenges of informed consent in high-stakes, Randomized oncology trials: A systematic review. MDM Policy Pract..

[18] Davis TC, Berkel HJ, Holcombe RF, Pramanik S, Divers SG (1998). Informed consent for clinical trials: A comparative study of standard versus simplified forms. J. Natl. Cancer Inst..

[19] National Cancer Institute. *Cancer Stat Facts: Cancer of Any Site.* https://seer.cancer.gov/statfacts/html/all.html.

[20] Rikard RV, Thompson MS, McKinney J, Beauchamp A (2016). Examining health literacy disparities in the United States: A third look at the National Assessment of Adult Literacy (NAAL). BMC Public Health.

[21] Kirsch, I., Jungeblut, A., Jenkins, L., & Kolstad, A. *Adult Literacy in America: A First Look at the Findings of the National Adult Literacy Survey*. https://nces.ed.gov/pubs93/93275.pdf (National Center for Education Statistics, U.S. Department of Education, 1993).

[22] Kutner, M., Greenberg, E., Jin, Y., & Paulsen, C. *The Health Literacy of America’s Adults: Results from the 2003 National Assessment of Adult Literacy*. https://nces.ed.gov/pubs2006/2006483.pdf (National Center for Education Statistics, U.S. Department of Education, 2006).

[23] Liang L, Brach C (2017). Health Literacy Universal Precautions are still a distant dream: Analysis of U.S. data on health literate practices. Health Literacy Res. Pract..

[24] National Academies of Sciences, Engineering, and Medicine. *Health Literacy in Clinical Research: Practice and Impact: Proceedings of a Workshop*. 10.17226/25616 (National Academies Press, 2020). 32484634

[25] MacLennan DL, Plahovinsak JL, MacLennan RJ, Jones CT (2023). Clinical trial site perspectives and practices on study participant diversity and inclusion. Clin. Pharmacol. Ther..

[26] Office for Human Research Protections. *Read the Belmont Report*. HHS.gov. https://www.hhs.gov/ohrp/regulations-and-policy/belmont-report/read-the-belmont-report/index.html (2018).

[27] Hadden KB (2017). Improving readability of informed consents for research at an academic medical institution. J. Clin. Transl. Sci..

[28] Hillyer GC (2020). Readability of cancer clinical trials websites. Cancer Control..

[29] Rampey, B. *et al*. *Skills of U.S. Unemployed, Young, and Older Adults in Sharper Focus: Results from the Program for the International Assessment of Adult Competencies* (*PIAAC*) *2012/2014: First Look*. https://nces.ed.gov/pubs2016/2016039rev.pdf (National Center for Education Statistics, U.S. Department of Education, 2016).

[30] Paasche-Orlow MK, Schillinger D, Greene SM, Wagner EH (2006). How health care systems can begin to address the challenge of limited literacy. J. Gen. Intern. Med..

[31] U.S. General Services Administration. *Law and Requirements*. plainlanguage.gov. https://www.plainlanguage.gov/law/ (2024).

[32] Cromley JG, Azevedo R (2007). Self-report of reading comprehension strategies: What are we measuring?. Metacognit. Learn..

[33] Just MA, Carpenter PA (1992). A capacity theory of comprehension: Individual differences in working memory. Psychol. Rev..

[34] Landi N (2009). An examination of the relationship between reading comprehension, higher-level and lower-level reading sub-skills in adults. Read. Writing.

[35] McCarthy KS, McNamara DS (2021). The multidimensional knowledge in text comprehension framework. Educ. Psychol..

[36] Okonski, G. *VT Writer. VisibleThread*. https://www.visiblethread.com/vt-writer/. Accessed 10 July 2022 (2022).

[37] MacGinitie, W., MacGinitie, R., Cooter, R., & Curry, S. Assessment: Gates-MacGinitie reading tests, third edition. *Read. Teacher* **43**(3), 256–258 (2023).

[38] Woodcock RW, Flanagan DP, Genshaft JL, Harrison PL (1997). The Woodcock-Johnson tests of cognitive ability—Revised. Contemporary Intellectual Assessment: Theories, Tests, and Issues.

[39] U.S. Food and Drug Administration. *2020 Drug Trials Snapshots Summary Report*. https://www.fda.gov/media/145718/download (2021).

[40] Virostko J, Capasso A, Yankeelov TE, Goodgame B (2019). Recent trends in the age at diagnosis of colorectal cancer in the US National Cancer Data Base, 2004–2015. Cancer.

[41] .Duma, N., Aguilera, J.V., Paludo, J. *et al*. Representation of minorities and women in oncology clinical trials: Review of the past 14 years. *JCO Oncol. Pract*. **14**, e1–e10 (2018). (Snow, C. *Reading for Understanding: Toward an R&D Program in Reading Comprehension*. https://www.rand.org/pubs/monograph_reports/MR1465.html, 2002). 10.1200/JOP.2017.025288 29099678

[42] Steen-Baker AA (2017). The effects of context on processing words during sentence reading among adults varying in age and literacy skill. Psychol. Aging.

[43] Barton AJ (2018). Health literacy: Essential for a culture of health. J. Contin. Educ. Nurs..

[44] Gesualdo F (2021). Digital tools in the informed consent process: A systematic review. BMC Med. Ethics.

[45] Seidel, E., Cortes, T., & Chong, C. *Digital Health Literacy*. https://psnet.ahrq.gov/primer/digital-health-literacy (Patient Safety Network, 2023).

[46] US Dept of Health and Human Services. *Think Cultural Health*. National CLAS Standards. https://thinkculturalhealth.hhs.gov/clas.

[47] Feinberg, I., Greenberg, D. & Talwar, A. Cultural competency in health literacy for older adults. In *Health Literacy Among Older Adults* (Kopera-Frye, K. Ed). Chap. 17. (S4 Carlisle Publishing, 2017).

[48] O'reilly T, McNamara DS (2007). Reversing the reverse cohesion effect: Good texts can be better for strategic, high-knowledge readers. Discourse Process..

[49] McCarthy KS, McNamara DS (2021). The multidimensional knowledge in text comprehension framework. Educ. Psychol..

