# Supplementary material for: Simplifying informed consent as a universal precaution
Source: Sci Rep. 2024 Jun 8;14:13195. doi: 10.1038/s41598-024-64139-9 (PMC11162480; doi:10.1038/s41598-024-64139-9)
Supplement: Supplementary file 1 — Supplementary Information. [file 41598_2024_64139_MOESM1_ESM.docx]

True/False Questions after Reading Informed Consent

Purpose of the Study

1. When I began my current cancer therapy, I was also agreeing to be in a clinical trial.
2. All the treatments and procedures in my clinical trial are standard for people with my type of cancer.
3. One of the researcher’s main purposes is to test how well two drugs work to treat colorectal cancer.
4. This document will tell me about risks and benefits from being in the study.
5. I do not have the option to talk with friends or family about this trial before I sign it.
6. The treatment being researched in this clinical trial has been proven to be the best treatment for my type of cancer.

Costs to be in the Study

1. I will be responsible for all costs related to being in this study.
2. There are things being done only for research that I will not have to pay for.

Side Effects

1. I may get both common and serious side effects from taking the drugs in this study.
2. Feeling nauseous or sick to my stomach is a common side effect of both drugs that will be used in this trial.
3. No one will be able to help me if I get side effects.
4. Doctors know what the side effects are from combining both drugs.

Stop Being in the Study

1. I will have to remain in the clinical trial even if I decide someday that I do not want to be in it anymore.
2. I do not have to return any of the leftover study drug if I leave the clinical trial.
3. Any study samples collected will be returned to me if I decide to leave the study.
